# *Leptospira* in Slaughtered Fattening Pigs in Southern Italy: Serological Survey and Molecular Typing

**DOI:** 10.3390/ani12050585

**Published:** 2022-02-25

**Authors:** Giusi Macaluso, Alessandra Torina, Valeria Blanda, Annalisa Guercio, Antonio Lastra, Ilenia Giacchino, Rosalia D’Agostino, Carmela Sciacca, Mario D’Incau, Cristina Bertasio, Francesca Grippi

**Affiliations:** 1 Istituto Zooprofilattico Sperimentale della Sicilia “A. Mirri”, 90129 Palermo, Italy; giusi.macaluso@izssicilia.it (G.M.); alessandra.torina@izssicilia.it (A.T.); annalisa.guercio@izssicilia.it (A.G.); antonio.lastra@izssicilia.it (A.L.); ileniajak2@gmail.com (I.G.); rosalia.dagostino1@gmail.com (R.D.); sciacca.carmela@gmail.com (C.S.); francesca.grippi@izssicilia.it (F.G.); 2 Centro di Referenza Nazionale per la Leptospirosi, Istituto Zooprofilattico Sperimentale della Lombardia e dell’Emilia Romagna “Bruno Ubertini”, 25124 Brescia, Italy; mario.dincau@izsler.it (M.D.); cristina.bertasio@izsler.it (C.B.)

**Keywords:** leptospirosis, MAT, real-time PCR, genotyping, pigs, Sicily

## Abstract

**Simple Summary:**

In this study, serological and molecular assays in 55 pigs in Sicily were conducted in order to investigate *Leptospira* spp. prevalence and to carry out strain characterization. A seropositivity rate of 16.4% was determined; 3.64% of kidney samples tested positive for pathogenic Leptospiral DNA. Obtained data showed that *Leptospira* infection is common among pigs in southern Italy, confirming the importance of Leptospiral infection in pigs and reaffirming the potential role of these animals as a source of infection for humans (occupational risk) and other animals. Our study delivers a comprehensive overview based on up-to-date data to deepen the knowledge of swine leptospiral infections, characterize new potential emerging strains, and reinforce control measures able to reduce the infection risk in swine herds.

**Abstract:**

Leptospirosis is a re-emerging zoonosis of worldwide significance; a wide spectrum of wild and domestic animal species act as natural or accidental hosts. Swine can act as maintenance or accidental hosts of pathogenic *Leptospira* spp. This study aimed at investigation of *Leptospira* spp. prevalence and diversity in slaughtered pigs in southern Italy (Sicily). In total, 55 samples of kidneys and blood were collected. Microscopic agglutination test and real-time PCR were performed to detect pathogenic and intermediately pathogenic *Leptospira*. Partial *rpoB* gene sequencing and multi-locus sequence typing (MLST) were performed to characterize *Leptospira* species. The analysis showed a seropositivity rate of 16.4%, with Australis representing the most frequently identified serogroup (63.64%); Pomona and Sejroe were detected with a prevalence of 27.27% and 9.09%, respectively. Pathogenic Leptospiral DNA was detected in 2 kidney samples (3.64%). *Leptospira* were identified through MLST as *L. borgpetersenii* serovar Tarassovi (serogroup Tarassovi). Obtained data confirmed the presence of *Leptospira* infection among pigs in southern Italy, suggesting that management of these animals may be considered an occupational risk for humans.

## 1. Introduction

Leptospirosis is a zoonosis occurring worldwide, caused by pathogenic spirochaetes of the genus *Leptospira*, transmitted through direct contact with the urine of infected animals or a urine-contaminated environment. It has a negative economic impact on farm animals, causing economic losses and serious human diseases and mortality [1].

The genus *Leptospira* contains 64 named species [2]. *Leptospira* have been classified serologically into more than 250 serovars [1,2,3,4]. Leptospires persist for a long time in the kidneys and genital tracts of domestic animals, including pigs, with intermittent shedding in the urine. This causes infections in humans and other animals [5,6,7]. Animal infections are caused by serovars maintained by the same or other species sharing the same geographical location [7].

Swine infections are caused by these pathogenic species: *L. borgpetersenii* (serovars Sejroe and Tarassovi), *L.interrogans* (serovars Pomona, Icterohaemorrhagiae, Canicola, and Bratislava), and *L. kirschneri* (serovars Grippotyphosa and Mozdok). Infections of *L. kirschneri* serovar Mozdok have been reported in pigs in various European countries [8,9,10] including Italy [11], and this serovar has been shown to be pathogenic for pigs, causing abortion and stillbirth in swine [12]. Serovars Bratislava and Pomona are uniquely adapted to swine; the others occasionally infect swine, being maintained in other species [13]. *L. interrogans* serovar Hardjo infects pigs sharing the same habitats with cattle. *L. interrogans* serovar Bratislava is the most frequent swine strain, with a doubtful role as a cause of disease [14].

Porcine leptospirosis imposes economic losses on pig farms, causing abortion, stillborn and weak piglets, and deaths soon after birth [15]. Leptospires cause serious illnesses depending on the serovar and the animal age [16]. When the infective agent enters a farm, its spread is rapid, mostly among fattening pigs [17].

In Italy, swine have been shown to maintain serovar Pomona (Pomona serogroup) and serovar Bratislava (Australis serogroup); serovar Tarassovi has been shown to be responsible for incidental infections [17]. Until 2010, a trivalent vaccine against these serogroups was available, but it was utilized by few swine farmers. In 2011, vaccinations were completely abandoned, due to poor understanding of the risk of leptospirosis and because of the treatments for more virulent diseases [11]. 

The Office International des Epizooties (OIE) reports the microscopic agglutination test (MAT) as the serological gold standard method [18]. The selection of antigens should include the serogroup strains circulating in the study area and those known to be maintained by the species to be analyzed [11].

Besides the classical conventional reference methods, over the years, several real-time polymerase chain reaction (PCR) methods and molecular typing techniques have been developed to directly investigate *Leptospira* DNA in biological samples, to examine individual genomic profiles and to investigate the epidemiology [11,19,20,21,22,23,24,25]. They provide diagnostic advantages, such as reduced turnaround times, low risk of contamination and greater sensitivity and specificity [25].

Following a protocol published by Weiss et al., 2016 [26], multilocus sequence typing (MLST) avoids pathogen isolation, since it can be directly performed on the biological sample DNA.

The data provided by serological and molecular investigations in the present study will be useful to characterize circulating strains and new emerging potential ones among pigs and to gain insight into the prevalence and epidemiology of porcine leptospirosis in southern Italy (Sicily), in order to increase specific control measures able to reduce the infection risk in pig farms.

## 2. Materials and Methods

### 2.1. Sample Composition

Between April and June 2019, sera, whole blood and kidney specimens were randomly collected from a total of 55 autochthonous healthy fattening pigs in a slaughterhouse in the province of Messina (Sicily, Italy), belonging to 5 Sicilian farms in the province of Messina and Palermo.

Blood samples were centrifugated at 845× *g* for 10 min at room temperature; sera were kept at 4 °C and kidney samples at −20 °C until use.

### 2.2. Serological Test

#### Microscopic Agglutination Test (MAT)

OIE guidelines were followed to perform the MAT [18,27], a serological test detecting antibodies to specific serovars using live leptospiral antigens. The strains, provided by the Italian National Reference Centre for Leptospirosis, were grown in liquid *Leptospira* Ellinghausen–McCullogh–Johnson–Harris (EMJH) culture medium for 4–8 days at 30 °C. The panel of antigens consisted of eight serogroups, representative of all the serogroups known to exist in Italy (*L. interrogans* serogroup Australis serovar Bratislava, *L. interrogans* serogroup Pomona serovar Pomona, *L. kirschneri* serogroup Grippotyphosa serovar Grippotyphosa, *L. borgpetersenii* serogroup Ballum serovar Ballum, *L. interrogans* serogroup Sejroe serovar Hardjo, *L. borgpetersenii* serogroup Tarassovi serovar Tarassovi, *L. interrogans* serogroup Icterohaemorragiae serovar Copenhageni, and *L. interrogans* serogroup Canicola serovar Canicola). The antigen–antibody complexes were assessed by dark-field microscopy. Samples showing titers equal to or higher than the MAT cut-off of 1:100 against one or more serovars were considered positive; the dilution of serum showing 50% agglutination was the endpoint.

### 2.3. Molecular Tests

#### 2.3.1. Real-Time PCR and PCR Investigations

For DNA extraction from kidney, the surface was flamed and 1 g of tissue withdrawn and homogenized in 9 mL of sterile physiological solution with Stomacher^®^ 80 Biomaster (Seward Limited, London, UK).

DNA was extracted from 0.2 mL of blood or homogenized kidney using the PureLink Genomic DNA kit (Invitrogen, Paisley, UK), by adding an internal control DNA (0.1 µL of per µL of elution volume) before the extraction.

A multiplex real-time PCR assay targeting *Leptospira* genus specific 16S ribosomal RNA gene (*rRNA* gene) and the pathogen specific *LipL32* gene on the external membrane of pathogenic *Leptospira,* was performed to detect intermediately pathogenic and pathogenic leptospires, respectively [22,28], by using Quantifast Pathogen + IC Kit (Qiagen, Hilden, German). The mix was composed of 5 µL of 5× Mastermix Quantifast, 2.5 µL of Internal Control assay, 700 nM of primers and 200 nM of the probe for *LipL32*, and 500 nM of primers and 150 nM of the probe for *16S rRNA*, in a 25 µL total volume. The assay was performed on a Bio-Rad CFX96 machine using DNA extracted from *Leptospira interrogans* serogroup Australis serovar Bratislava, kindly supplied by Istituto Zooprofilattico Sperimentale (IZS) of Lombardia and Emilia Romagna (IZSLER) as positive control. The following thermal conditions were used: 95 °C for 5 min, 45 cycles of 95 °C for 15 sec and 60 °C for 30 sec [27].

Partial *rpoB* gene sequencing was performed to characterize *Leptospira* species [29]. The assay was performed using the GoTaq^®^ G2 DNA Polymerase (Promega Corporation, Milan, Italy) in a 25 µL reaction mix, using 5 µL of extracted DNA, 5 µL of 5× GoTaq^®^ Reaction Buffer, 1 µL of a dNTP mix (200 µM), 0.6 µL of each primer (0.5 µM), and 0.125 µL of GoTaq^®^ G2 DNA Polymerase. The following thermal conditions were used: 95 °C for 2 min to activate TaqPol followed by 35 cycles of 94 °C for 30 s, 51 °C for 30 s, 72 °C for 30 s, and a final extension of 72 °C for 7 min. The amplification products were sequenced by BMR Genomics, Padova, Italy; the analysis were carried out using BioEdit Software [30].

Table 1 shows the sequences of primers and probe employed for molecular analysis. Confidence intervals (CI_95%_) of the positive results were calculated for proportions.

#### 2.3.2. MLST Analyses

Real-time PCR positive samples were sent to the Italian Reference Centre for Animal Leptospirosis (IZSLER, Brescia) for MLST genotyping analyses [11]. The scheme proposed by Boonsilp et al., 2013 [31], based on sequencing of seven housekeeping genes, was employed. Allele numbers and pattern profiles were queried against the Bacterial Isolate Genome Sequence Database (BIGSdb) (available online: https://pubmlst.org/Leptospira/) (accessed on 6 February 2021) to identify the infecting strain.

Sequences of the seven MLST genes were concatenated (final sequence of 3111 nucleotides) and aligned with nucleotide sequences of reference strains present in the collection of Italian Reference Centre for Animal Leptospirosis using BioEdit software version 4.0 (available online: www.mbio.ncsu.edu/BioEdit/bioedit.html (accessed on 6 February 2021)). A phylogenetic analysis was conducted in MEGA X [32] using the neighbor-joining method and the maximum composite likelihood model [33] with a bootstrap analysis of 1000 replicates.

## 3. Results

### 3.1. Microscopic Agglutination Test (MAT)

Overall, 9 out of 55 sera collected were positive based on MAT, with a seropositivity of 16.4% (CI_95%_ 0.07–0.26 %) (cut-off ≥ 1:100). 

The most frequently identified serogroup was Australis (12.73%, CI_95%_ 0.04–0.21% of the total samples, 63.64%, CI_95%_ 0.35–0.92% of the positive samples), followed by Pomona (5.45% CI_95%_ −0.01–0.11% of the total samples and 27.27%, CI_95%_ 0.01–0.54% of the positive samples) and Sejroe (1.82% CI_95%_ −0.02–0.05% of the total samples and 9.09%, CI_95%_ −0.08–0.26% of the positive samples). Among the positive samples, 7 (12.73% CI_95%_ 0.04–0.21%) of the total samples and 77.77% CI_95%_ 0.51–1.05% of the positive samples, tests positive for one serogroup, and 2 (3.64% CI_95%_ 0.04–0.96% of the total samples and 22.22% CI_95%_ 0.22–0.78% of the positive samples) were positive for more serogroups (combination represented by Australis-Pomona) (Table 2).

The MAT titers of the single positive samples were mostly low, except for the serogroup Sejroe (showing 1:400 titer). In particular, 85.71% (CI_95%_ 0.39–0.97%) of the samples positive for the serogroup Australis and all of those positive for Pomona showed low antibody titers (Table 3).

### 3.2. Molecular Investigation and Genotyping Analyses

By multiplex real-time PCR, pathogenic Leptospiral DNA was detected in 2 out of 55 kidneys (3.64%, CI_95%_ −0.01–0.08%). No blood samples tested positive.

Partial rpoB gene sequencing, carried out for Leptospira genotype assignment, yielded negative results, probably due to both a lower sensitivity of the test and to the low amount of pathogen DNA.

Samples of the two pigs that tested positive to leptospiral DNA were submitted for MLST analysis. A complete MLST profile was obtained from one pig (ID: Kidney 21_2019), while for the other pig (ID: Kidney 20_2019) a partial profile was defined (Table 4).

**Figure 1:**
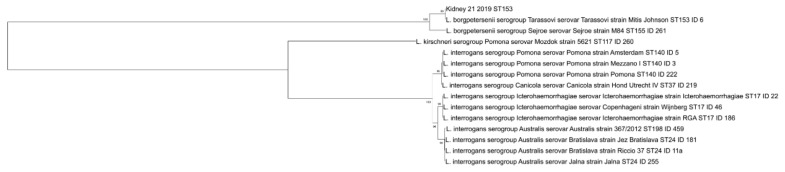
Phylogenetic tree based on concatenated sequences of the seven MLST genes. The DNA of kidney sample with a complete MLST profile is indicated with its progressive number and the year of sampling. The reference strains are indicated with their IDs, which represent a unique identification number of the strain present in the collection of Italian Reference Centre for Animal Leptospirosis. MEGA software was used for phylogeny using the neighbor-joining method. The percentages of replicate trees in which the associated taxa clustered together in the bootstrap test (1000 replicates) are shown next to the branches.

Both detected *Leptospira* belonged to ST153 that clustered with reference strains characterized as L. *borgpetersenii* serovar Tarassovi (serogroup Tarassovi) from the PubMLST and Italian Reference Centre for Animal Leptospirosis (IZSLER, Brescia, Italy) databases (Figure 1).

Kidney 21_2019 belonged to an animal showing antibodies against Australis serogroup, with a 1:200 MAT titer. Kidney 20_2019 was collected from a serologically negative animal using MAT.

## 4. Discussion

The main objective of this study was to estimate the seroprevalence of *Leptospira* antibodies and the identification by molecular investigations of infective *Leptospira* serovars in slaughter pigs in southern Italy (Sicily). In Italy, the fifth largest European producer of pig meat [34], about 14 million pigs are slaughtered for meat each year, and over 4000 people work in the pork production chain [35]. Leptospirosis is not included in the OIE list of notifiable animal diseases, but it is currently considered a notifiable infection in Italy [36], with consequent significant economic losses, because affected farms are subject to seizure and restrictive controls are applied.

Control and prevention of leptospirosis in pigs requires a combination of different strategies intended to improve husbandry practices, prevent animal infection, and protect humans, as well as promote vaccination [37,38].

In this study, the MAT test conducted on 55 pigs in Sicily showed a seropositivity of 16.4%, considering single and multiple positive sera, thus being partially in agreement with other previous studies [11,39,40]. Bertelloni [39] confirmed the seroprevalence identified in this study, reporting a seroprevalence of 16.6% among slaughtered pigs in north-central Italy; Bertasio et al. [11] conducted a similar study in northern Italy, detecting a slightly lower seroprevalence of 13.05% in fattening pigs, with Australis the most frequently identified serogroup, followed by Pomona, Tarassovi and Icterohaemorrhagie. In a study by Cerri et al. [40], a lower prevalence (8.85%) was detected in Italian swine sera, using a cut-off of 400, thus reducing the prevalence value compared to the cut-off of 100 used in the present study.

A high percentage of pigs positive for Australis serogroup (63.64%) followed by Pomona and Sejroe (27.27% and 9.09%, respectively) was observed. A similar study [17] showed that Pomona, Tarassovi, Bratislava and Muenchen are the most common serovars among swine in Italy. These data indicate swine can act as a reservoir host for these serogroups and that Australis is mainly present in pigs in southern Italy, confirming results from many regions worldwide [16]. A study conducted in five provinces in Vietnam showed a seroprevalence of 8.17% among fattening pigs [41]. 

The detection of serogroups by MAT depends on the investigation phase [42]; the induction of low antibody titers against common antigens of *Leptospira* spp., as well as cross-reactions of serogroups, are typical of the first phase of infection [42,43]. Titers of 1:100 or 1:200 may be suggestive of an early stage of infection; higher titers can be considered distinctive of endemic infection [44]. The low titers observed in this study in most samples could suggest a recent exposure to *Leptospira* spp. Moreover, the presence of positive sera reactions, at the same time, with two serovars (Australis-Pomona), indicated cross-reactions and confirmed the first phase of infection, the latter because the induction of antibodies against common antigens of *Leptospira* is frequent during the acute phase of infection [11]. It has been shown that serovar Mozdok infection causes serological cross-reactions with the Australis, Icteroahemorrhagiae and Grippotyphosa serogroups [12].

In this study, for the sample Kidney 21_2019, a co-infection of Australis and Tarassovi serogroups could be hypothesized, because the animals came from farms where the simultaneous presence of different strains could have been possible. Indeed, while antibodies against Australis serogroup persisted over time with low titers, Tarassovi-specific antibodies were no longer detectable at the time of blood collection. It is also particularly difficult to detect the Australis serogroup in the kidney of fattening animals [45].

In regions where vaccination against leptospirosis has been practiced, including China, Japan, Cuba, and Europe, declines in overall seroprevalence have been reported [34]. This decrease has also been connected with improved housing, limiting interactions between animals and the environment [37]. A study conducted in Greece reported a seroprevalence of 17.8% in pig farms [46].

Only two kidneys tested positive by real-time PCR. One of them was collected from a serologically negative animal, and the second one belonged to an animal showing antibodies against the Australis serogroup with a 1:200 MAT titer. A complete MLST profile was obtained from this latter kidney, while for the other one, a partial profile was defined.

The MLST confirmed the circulation of the Tarassovi serogroup, rarely detected and isolated by serological tests. The isolation of Tarassovi reported in the present study supports the hypothesis that pigs could act as a reservoir for this serogroup [47,48].

A study conducted in Sicily showed an high prevalence of leptospires among free-roaming semi-wild black swine, and this was attributed to their wild nature [49].

The percentages of positivity observed in Sicily compared with the other analyzed regions could be due to particular environmental conditions, potential risk factors and the abundance of reservoirs in the wild fauna

More recent studies in Europe have reported an increase in leptospirosis associated with wetter climatic conditions, promoting the prolonged environmental survival of *Leptospira* bacteria. Moreover, new climatic conditions have induced a change in herd management in Italy, increasing outdoor activities to improve animal welfare [39,44,50]. In the farms of origin, the bacteria could have been transiently present in water streams, rivers and small pools shared between swine and wildlife, and the pigs could have shared watering spots with the rich local wild fauna (wild pigs, wild boars, foxes, martens, etc.). Among reservoirs, wild boar (*Sus scrofa*), as well as all swine, are considered the well-known maintenance host to the Tarassovi *Leptospira borgpetersenii* serogroup and Pomona and Australis *Leptospira interrogans* serogroups [13]. Moreover, due to their population abundance in all European countries, this animal species could be a suitable indicator of *Leptospira* prevalence in a specific area and a potential source of leptospires that then infect humans and domestic animals [51,52,53,54].

Because of their genetic relationship to domestic swine, wild boars play an important role in the transmission of leptospirosis among free living and domestic species [55] and could be identified as a potential source of infection for domestic pigs [56,57], as well as humans [57].

Different studies conducted across Europe on wild boars have shown variable seroprevalence of *Leptospira* from 65.4% in Portugal, [53], 45.8% in Slovenia [46] and 31.9% in Croatia [58], to 2.6% in Italy [59] and 3.1% in Sweden [60]. This variation across regions may be due to differences in the populations of wild small mammals acting as maintenance hosts [23].

Slaughterhouses occasionally represent an important surveillance station, mainly for foodborne pathogens (*Salmonella*, *Campylobacter* and *Trichinella*). They can also allow the detection of specific swine infections [50]. Moreover, in order to control *Toxoplasma gondii* infections in the pork supply chain, recommended measures developed by the European Food Safety Authority (EFSA) include serological testing of pigs for this pathogen at the farms or slaughterhouses and on-farm audits for risk factors associated with this infection [61,62]. For these reasons, slaughterhouses could assume an important epidemiological role in highlighting some important zoonosis not detected in the farms. Moreover, the distribution of serovars in slaughtered pigs could be assumed to reflect the distribution of serovars in pig farms.

Swine vaccination against *Leptospira* in Italy led to a decrease in this infection in the pig population [43]. Starting from 2011, vaccinations against *Leptospira* spp. were no longer practiced, and the management of the breeding herd was adopted as strategy. Strong surveillance systems could improve understanding of the disease epidemiology, and the application of rigorous biosecurity controls and an effective specific prevention strategy (vaccination, slaughterhouse screening) together with farm management could limit pathogen transmission in the herd.

A limitation of this study was the small sampling size. However, the results obtained could provide useful information about this zoonotic infection among pigs in Sicily and improve occupational awareness of the heightened exposure-related health risks to slaughterhouse workers due to poor use of protective devices and measures in the areas covered by this study [63].

## 5. Conclusions

The data obtained in this study confirmed the presence of *Leptospira* infection among pigs in southern Italy. It is important to use both serological and molecular diagnostic techniques complementarily to identify infected individuals. The serological survey evidenced that Australis and Pomona were the most common serogroups causing leptospirosis in pigs reared in Sicily. Furthermore, the molecular detection of *L. borgpetersenii* serovar Tarassovi (serogroup Tarassovi) as the genotype responsible for swine leptospirosis provided useful information to better understand the disease’s epidemiology and etiology. The control of zoonotic swine pathogens such as *Leptospira* spp. in slaughterhouses is important to reduce animal and human infections and to limit the related economic losses to farms. 

## Figures and Tables

**Table 1:** Molecular methods performed to detect and genotype *Leptospira* spp.

Molecular Method	Primers Probes	Target	PCR Product Length	Reference
Real Time PCR	LipL32-45F	*LipL32*	242 bp	Stoddard et al., 2009 Bedir et al., 2010
	LipL32-286R			
	LipL32-189P			
Real Time PCR	Lep-F	*16S rRNA*	173 bp	
	Lep-R			
	Lep-P			
Sequencing	Lepto 1900-F Lepto 2500-R	*rpoB*	600 bp	La Scola et al., 2006

**Table 2:** Numbers and percentages of serum samples testing positive, using MAT for Leptospira serogroups.

	Serogroup							
	A	B	C	G	I	P	S	T
N. of positive samples	7	/	/	/	/	3	1	/
Percentage (%) of the positives (*n* = 9)	63.64	/	/	/	/	27.27	9.09	/
Percentage (%) of the total (*n* = 55)	12.73	/	/	/	/	5.45	1.82	/

A, Australis; B, Ballum; C, Canicola; G, Grippotyphosa; I, Icterohaemorrhagiae; P, Pomona; S, Sejroe; T, Tarassovi.

**Table 3:** MAT titer distributions of positive sera reacting to one serogroup.

Serogroup	Titer			Total
	1:100	1:200	1:400	
Australis	6	1	/	7
Pomona	/	3	/	3
Sejroe	/	/	1	1

**Table 4:** Results of multi-locus sequence typing (MLST) analysis.

ID	ST	*glmU*	*pntA*	*sucA*	*tpiA*	*pfkB*	*mreA*	*caiB*
Kidney 20_2019	153 (partial)	29	35	n.d.	35	39	28	31
Kidney 21_2019	153	29	35	33	35	39	28	31

ST: sequence type; n.d.: not defined.

## Data Availability

The data that support the findings of this study are available from the corresponding author upon reasonable request.
